# Frequent PRAME Expression in Ameloblastoma: Immunohistochemical Profiling, Clinicopathologic Correlations, and Interobserver Reproducibility in a 37-Case Cohort

**DOI:** 10.3390/ijms27219771

**Published:** 2026-10-30

**Authors:** Fedor Popovic, Dusan Spasic, Novica Boricic, Srdjan Milanovic, Sofija Glumac, Branko Dozic, Boban Anicic

**Affiliations:** 1 Clinic for Maxillofacial Surgery, School of Dental Medicine, University of Belgrade, 11000 Belgrade, Serbia; drfedorpopovic@gmail.com (F.P.); boban944@gmail.com (B.A.); 2 Department of Pharmacology, Clinical Pharmacology and Toxicology, Faculty of Medicine, University of Belgrade, 11000 Belgrade, Serbia; dusan.spasic@beograd.com; 3 Institute of Pathology, Faculty of Medicine, University of Belgrade, 11000 Belgrade, Serbia; boricic.novica@gmail.com (N.B.); sofijaglumac09@gmail.com (S.G.); 4 Institute for Oncology and Radiology of Serbia, Clinic for Radiation Oncology, 11000 Belgrade, Serbia; srdjan_milanovitch@yahoo.com; 5 Department of Pathology, School of Dental Medicine, University of Belgrade, 11000 Belgrade, Serbia

**Keywords:** ameloblastoma, PRAME, immunohistochemistry, recurrence, odontogenic tumor, biomarker, interobserver agreement

## Abstract

Ameloblastoma is a histologically benign odontogenic epithelial tumor with locally destructive growth and a clinically relevant risk of recurrence. Established molecular research has been largely focused on MAPK-pathway alterations, whereas markers capturing other dimensions of tumor biology remain limited. Preferentially expressed antigen in melanoma (PRAME) is a tumor-associated antigen implicated in retinoic-acid receptor signaling, altered differentiation, and antitumor immune recognition, but dedicated data in ameloblastoma remain limited. In this retrospective cohort, 37 histologically confirmed ameloblastomas were evaluated immunohistochemically for PRAME on tissue microarray sections by two independent pathologists. A four-tier extent score (0, 1+, 2+, 3+) and a four-tier intensity score (absent, weak, moderate, strong) were recorded. PRAME positivity was defined as nuclear staining in ≥25% of tumor cells for exploratory association analyses. Clinicopathologic variables included age, sex, anatomic site, side, mandibular subsite, histologic type, surgical approach, recurrence, and follow-up. Interobserver reproducibility was assessed using percentage agreement and Cohen’s κ statistics. Holm adjustment was applied as a post hoc sensitivity analysis across eight exploratory clinicopathological comparisons. PRAME was positive in 30 of 37 tumors (81.1%; exact 95% CI 64.8–92.0). Twenty-nine tumors (78.4%) received a 3+ extent score from both observers, seven (18.9%) were negative, and the remaining tumor was scored 1+ by one observer and 2+ by the other. Extent scoring agreed in 36 of 37 cases (97.3% agreement; Cohen kappa = 0.923). Intensity scoring agreed in 35 of 37 cases (94.6%); unweighted kappa was 0.905, with linear and quadratic weighted kappa values of 0.927 and 0.949. PRAME positivity was more frequent in women than in men (95.2% vs. 62.5%; unadjusted two-sided Fisher exact *p* = 0.029; Holm-adjusted *p* = 0.230). All 11 patients with documented recurrence had PRAME-positive tumors, compared with 19 of 26 patients without documented recurrence (unadjusted *p* = 0.080). PRAME positivity was also more frequent among conservatively than radically treated patients (91.7% vs. 61.5%; unadjusted *p* = 0.072). None of the eight clinicopathological comparisons met the 0.05 threshold after Holm adjustment. PRAME is frequently and extensively expressed in ameloblastoma and can be assessed with excellent interobserver reproducibility. The recurrence and treatment patterns are hypothesis-generating, but the small cohort, sparse PRAME-negative subgroup, and treatment imbalance preclude claims of independent prognostic value. Larger multicenter studies with standardized scoring, molecular validation, and time-to-event analysis are warranted.

## 1. Introduction

Ameloblastoma is a histologically benign odontogenic epithelial tumor whose morbidity is driven by locally infiltrative growth rather than metastatic potential. It can expand through cancellous bone, perforate cortical boundaries, displace or resorb teeth and extend into adjacent soft tissues; recurrence remains significantly clinically important. Contemporary diagnosis and classification therefore integrate morphology with clinical and radiologic context and, increasingly, molecular findings [1,2]. Most tumors arise in the mandible, particularly at posterior sites, and treatment requires balancing durable local control against functional and aesthetic morbidity [3,4].

Recurrence risk is determined by tumor type, anatomic extent, completeness of removal, surgical strategy, and duration of follow-up. Conservative procedures preserve mandibular continuity and reduce immediate morbidity, but pooled evidence shows higher recurrence rates for conventional intraosseous ameloblastoma than after radical resection [5,6]. Histologic pattern alone does not reliably identify which individual tumor will recur, creating interest in biomarkers that capture biologic behavior beyond morphology. Any candidate marker must, however, first show a reproducible staining pattern before prognostic claims can be considered.

Molecular studies implicate matrix remodeling, osteoclastogenic signaling, growth-factor networks, and developmental pathways in the locally invasive phenotype [7,8]. Activating alterations in the FGFR2-RAS-BRAF/MAPK axis are particularly prominent, and BRAF V600E mutation is frequent in mandibular tumors [9,10]. Responses to BRAF-directed therapy in selected locally advanced tumors support the clinical relevance of this pathway [11], whereas an association between BRAF status and recurrence has not been consistently demonstrated [10]. These observations provide a rationale for examining additional expression programs while distinguishing exploratory immunophenotypic biomarkers from molecularly confirmed actionable alterations.

PRAME (preferentially expressed antigen in melanoma) is a tumor-associated antigen commonly grouped with cancer-testis antigens. PRAME can repress retinoic-acid receptor signaling, thereby attenuating retinoic-acid-mediated differentiation, growth arrest, and apoptosis [12]. In melanocytic pathology, diffuse nuclear expression can support melanoma in an appropriate morphologic context [13]; however, PRAME expression occurs across many epithelial and nonepithelial neoplasms and is not lineage-specific [14]. Its restricted expression in most normal adult tissues and its immunogenicity have also prompted PRAME-directed therapeutic research [15]. Dedicated data on its expression and scoring reproducibility in ameloblastoma remain limited.

The primary objective of this study was to characterize the prevalence and distribution of PRAME immunohistochemical expression in a retrospective cohort of ameloblastomas. A secondary objective was to quantify interobserver reproducibility for extent and intensity scoring. Exploratory objectives were to examine associations between PRAME status and demographic, anatomic, histologic, treatment, and recurrence variables. Because the cohort was small and the PRAME-negative subgroup was sparse, no independent prognostic validation was attempted and all association analyses were interpreted as hypothesis-generating.

## 2. Results

### 2.1. Clinicopathologic Characteristics

The cohort comprised 37 patients with a mean age of 41.5 ± 18.9 years. Twenty-one patients (56.8%) were women and 16 (43.2%) were men. Median follow-up was 42 months (range, 2–120 months). Median follow-up was 39 months (range, 2–120 months) in the PRAME-positive group and 42 months (range, 3–120 months) in the PRAME-negative group. Thirty-three tumors (89.2%) arose in the mandible and four (10.8%) in the maxilla. Among mandibular tumors, 27 of 33 (81.8%) were posterior. Twenty-one tumors (56.8%) were left-sided.

Conventional ameloblastoma accounted for 34 cases (91.9%), including 31 follicular and three plexiform tumors. Three tumors (8.1%) were unicystic. Conservative surgery was performed in 24 patients (64.9%), whereas 13 (35.1%) underwent radical surgery; six of the radical procedures included reconstruction. Recurrence was documented in 11 patients (29.7%), repeat recurrence in one of those 11 patients, and metastasis in one patient (2.7%). The clinicopathological characteristics of the study cohort are summarized in Table 1.

### 2.2. Distribution of PRAME Expression

PRAME positivity was identified in 30 of 37 tumors (81.1%; exact 95% CI 64.8–92.0). All seven tumors assigned extent score 0 (18.9%) showed complete absence of nuclear PRAME staining. Twenty-nine tumors (78.4%) received a 3+ extent score from both observers. The remaining tumor was scored 1+ by Pathologist 1 and 2+ by Pathologist 2.

For staining intensity, both observers classified seven tumors (18.9%) as absent. Pathologist 1 classified one tumor as weak, eight as moderate, and 21 as strong; Pathologist 2 classified none as weak, seven as moderate, and 23 as strong. Thus, strong staining was the dominant intensity category for both observers.

**Figure 1:**
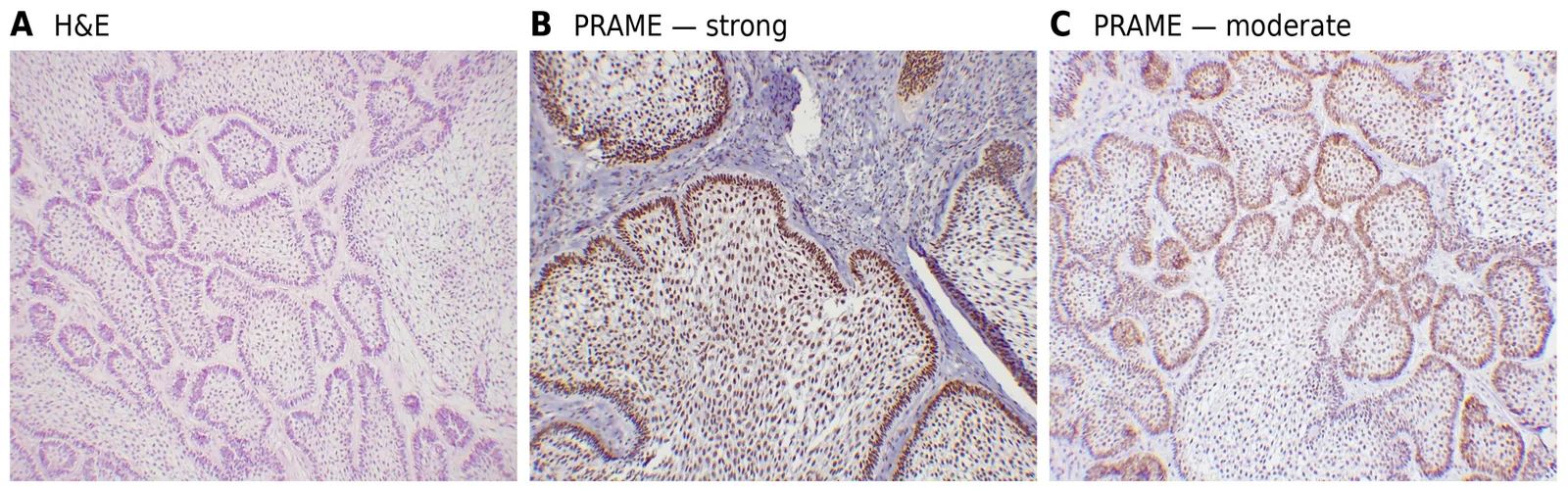
Representative histological features and PRAME immunoreactivity in ameloblastoma.

Representative histomorphology and examples of strong and moderate nuclear PRAME staining are illustrated in Figure 1.

**Figure 2:**
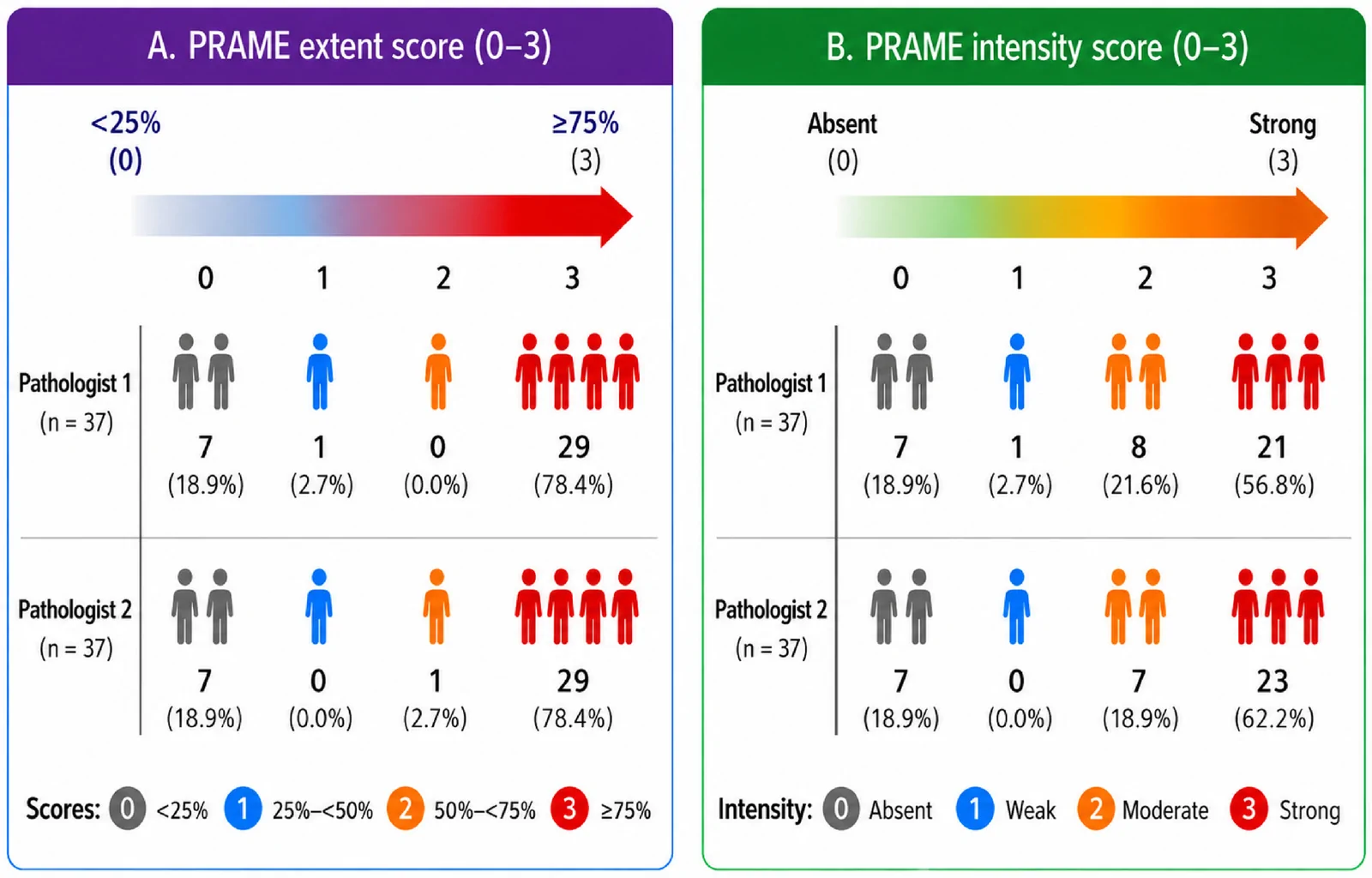
Distribution of PRAME immunohistochemical findings. (**A**) Four-tier extent scores assigned by the two pathologists. (**B**) Staining-intensity categories assigned by the two pathologists. Percentages are calculated using n = 37.

The distributions of PRAME extent and intensity scores are shown in Figure 2.

### 2.3. Interobserver Agreement

**Figure 3:**
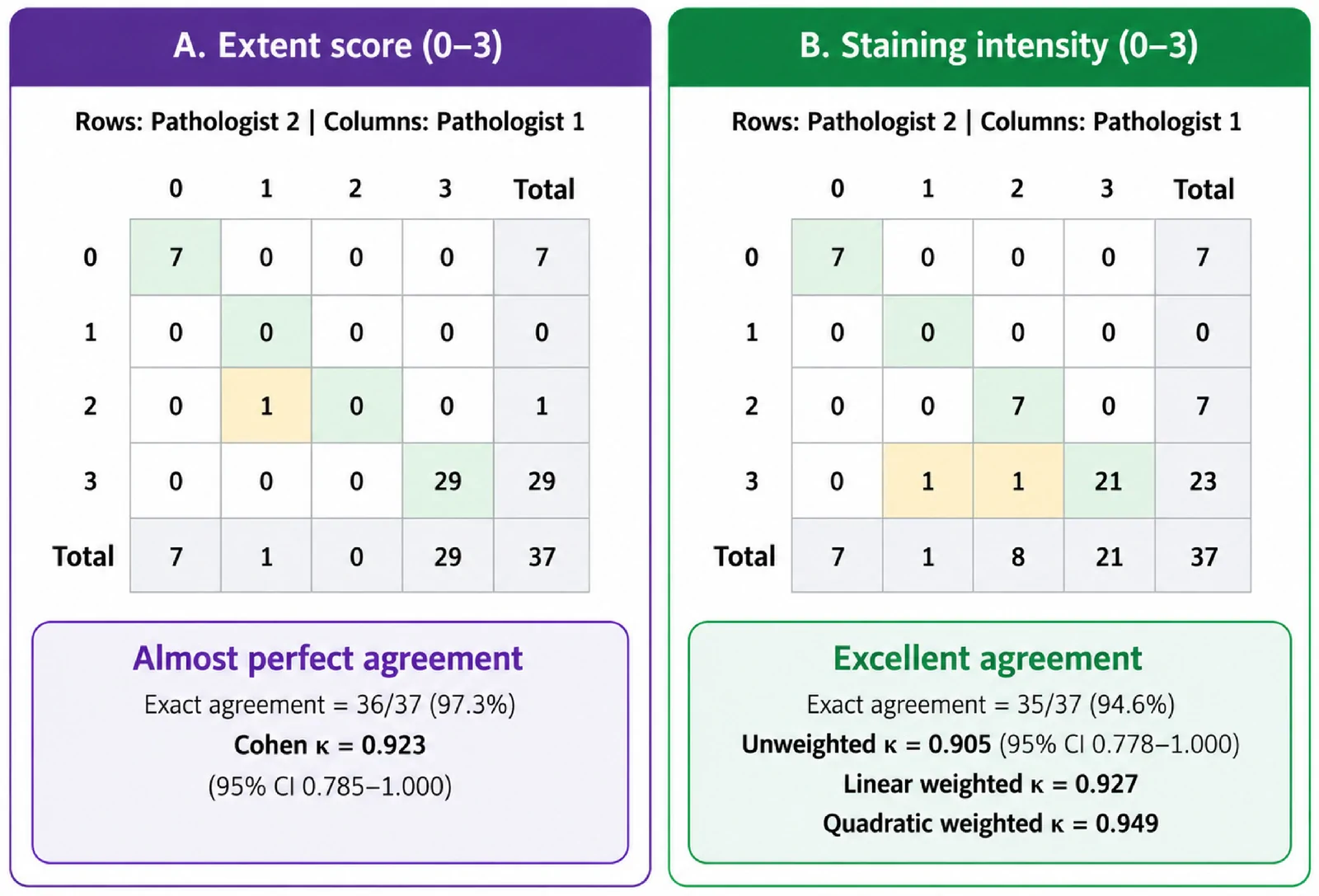
Interobserver agreement for PRAME assessment. (**A**) Confusion matrix for the four-tier extent score, showing agreement in 36 of 37 cases, with one 1+ versus 2+ discordance. (**B**) Confusion matrix for staining intensity, showing 35 concordant and two discordant cases.

The pathologists agreed on the PRAME extent score in 36 out of 37 cases (97.3%), corresponding to almost perfect agreement Cohen kappa = 0.923 (95% CI 0.785–1.000). The intensity assessment was also highly reproducible: 35 of 37 cases (94.6%) were classified identically. Unweighted Cohen kappa was 0.905 (95% CI 0.778–1.000), and the linear and quadratic weighted kappa values were 0.927 and 0.949, respectively. The two intensity disagreements were confined to one weak-versus-strong classification and one moderate-versus-strong classification; no disagreement involved the distinction between absent and present staining. Interobserver agreement statistics are summarized in Table 2 and illustrated by the confusion matrices in Figure 3. The individual paired extent and intensity scores underlying the agreement analyses are provided in Supplementary Table S1.

### 2.4. Exploratory Clinicopathologic Associations

PRAME positivity was observed in 20 of 21 women (95.2%) and 10 of 16 men (62.5%). The unadjusted odds ratio was 12.0 (95% CI 1.27–113.74), with a two-sided Fisher exact *p* value of 0.029. The Holm-adjusted *p* value across the eight clinicopathological comparisons was 0.230. The wide confidence interval and small PRAME-negative subgroup indicate substantial uncertainty in the magnitude of the association.

All 11 patients with documented recurrence had PRAME-positive tumors. Recurrence was documented in 11 of 30 PRAME-positive patients (36.7%) and none of the seven PRAME-negative patients (unadjusted *p* = 0.080; Holm-adjusted *p* = 0.506). PRAME positivity was also more frequent among conservatively treated patients than among radically treated patients (22/24, 91.7% vs. 8/13, 61.5%; unadjusted *p* = 0.072; Holm-adjusted *p* = 0.506).

**Figure 4:**
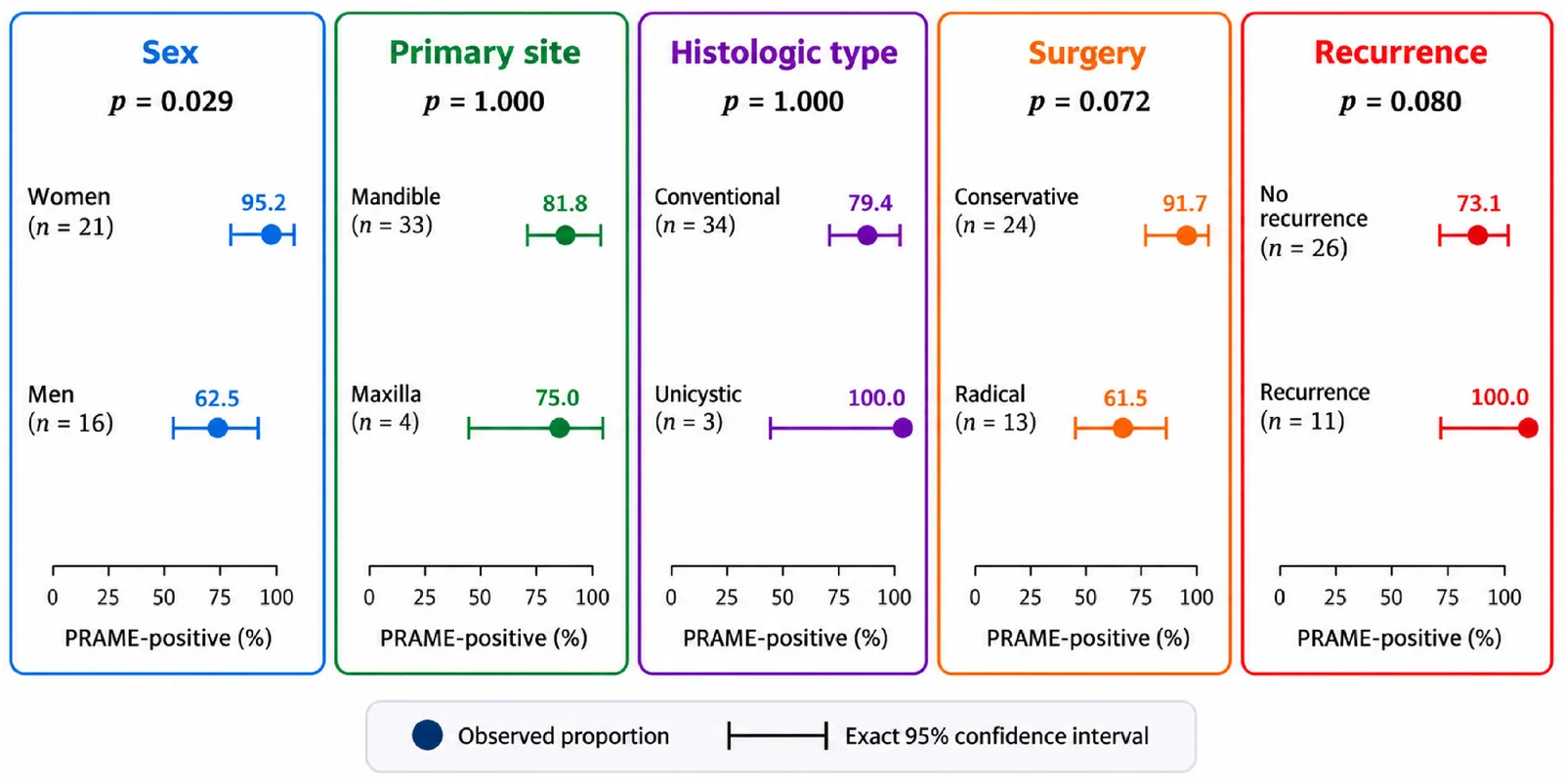
PRAME positivity across selected clinicopathological strata. Points show observed proportions and horizontal lines show exact binomial 95% confidence intervals. Displayed *p* values are unadjusted two-sided Fisher exact *p* values. Holm-adjusted *p* values for the eight clinicopathological comparisons are provided in Table 3. All comparisons are exploratory, and wide intervals reflect small subgroup sizes.

Mean age was 48.6 ± 16.2 years in the PRAME-negative group and 39.8 ± 19.3 years in the PRAME-positive group (unadjusted *p* = 0.276). The unadjusted *p* values for primary site, posterior mandibular localization, side, and histological type were 1.000, 0.295, 0.437, and 1.000, respectively. None of the eight clinicopathological comparisons met the 0.05 threshold after Holm adjustment (Table 3). Selected clinicopathological comparisons are illustrated in Figure 4.

To describe documented recurrence in relation to both PRAME status and surgical management, recurrence counts and proportions were jointly stratified by PRAME status and surgical approach (Table 4). Among conservatively treated patients, recurrence was documented in 10 of 22 PRAME-positive cases (45.5%) and neither of the two PRAME-negative cases. Among radically treated patients, recurrence was documented in one of eight PRAME-positive cases (12.5%) and none of the five PRAME-negative cases. These comparisons were descriptive, with small subgroup sizes and wide confidence intervals limiting their interpretation.

**Figure 5:**
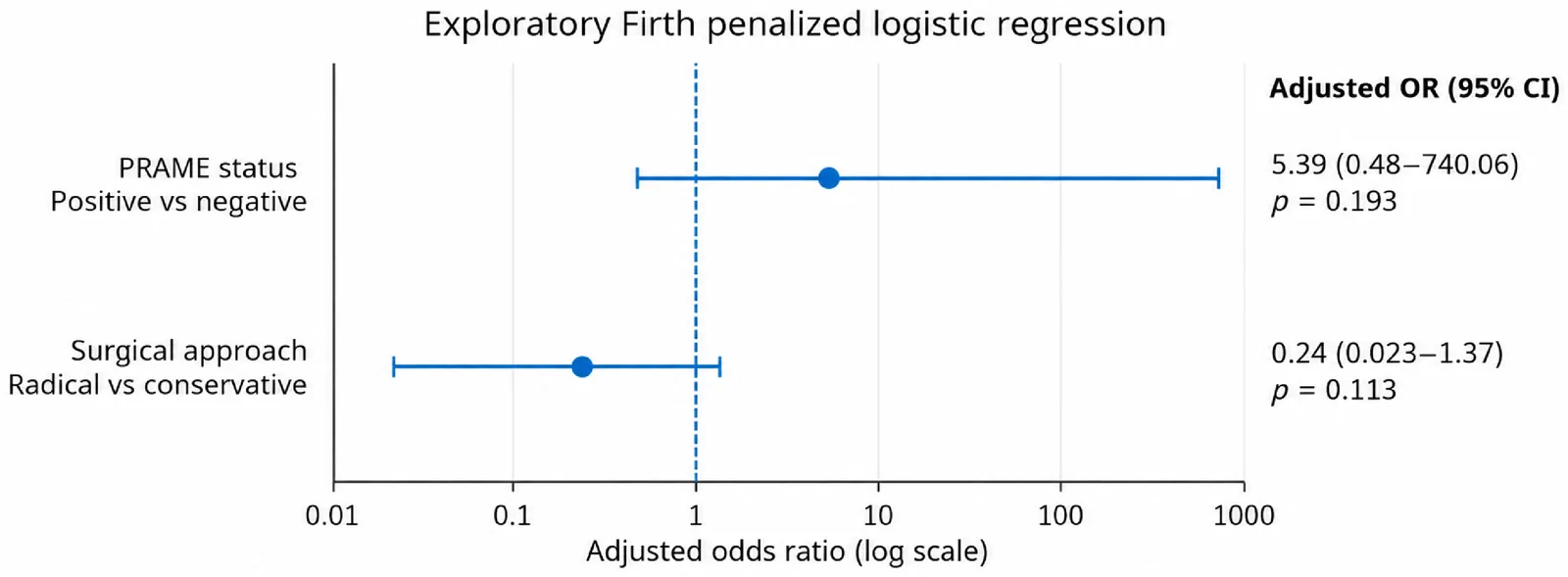
Forest plot illustrating adjusted odds ratios and 95% confidence intervals from the exploratory Firth penalized logistic regression analysis.

To further investigate the association between PRAME expression and documented recurrence while accounting for surgical management, an exploratory Firth penalized logistic regression analysis was performed (Figure 5). After adjustment for surgical approach, PRAME positivity was associated with an odds ratio of 5.39 for documented recurrence (95% profile penalized-likelihood CI: 0.48–740.06; *p* = 0.193). Radical surgery, adjusted for PRAME status, yielded an odds ratio of 0.24 (95% CI: 0.023–1.37; *p* = 0.113). Both estimates were imprecise, with confidence intervals crossing the null value. These findings highlight the substantial statistical uncertainty associated with the small cohort and do not establish an independent prognostic association.

## 3. Discussion

Three findings define this study. First, PRAME immunoreactivity was common (81.1%) and usually extensive, with 29 of 37 tumors assigned the 3+ extent category. Second, scoring was highly reproducible: extent assessment was almost perfect between observers and intensity assessment showed excellent agreement. Third, the observed differences by sex, recurrence, and treatment were exploratory and do not establish independent prognostic value. This hierarchy is important: the data support prevalence and reproducibility, whereas clinical prediction remains unvalidated.

The cohort broadly reflected the expected clinicopathologic profile of ameloblastoma. The mean age of 41.5 years, marked mandibular predominance, frequent posterior mandibular involvement, and predominance of conventional follicular tumors are consistent with large demographic and institutional series [3,16,17,18,19,20,21,22]. The observed recurrence frequency of 29.7% is plausible for a mixed cohort treated with conservative and radical approaches, although reported recurrence rates vary with tumor type, surgical method, margin assessment, referral pattern, and follow-up duration [5,20,23]. Meta-analytic evidence showing higher recurrence after conservative treatment [6,23,24] underscores why treatment must be considered whenever a biomarker is related to recurrence.

PRAME provides a biologically plausible, but unproven, link to tumor behavior. By repressing retinoic-acid receptor signaling, PRAME may attenuate differentiation, growth arrest, and apoptosis [12]. Ameloblastoma invasion is also associated with matrix metalloproteinases and growth factors [7,25], RANK/RANKL/OPG-mediated osteoclastogenesis [26,27], and aberrant Wnt signaling [28]. PRAME expression could therefore mark altered differentiation or survival signaling, but the present immunohistochemical study cannot establish causality or demonstrate direct interaction with these pathways.

PRAME should also be considered alongside protein markers previously investigated in ameloblastoma. Ki-67 has been widely used to assess proliferative activity in ameloblastoma [29], while the minichromosome maintenance proteins MCM2 and MCM3 have also been investigated as proliferation markers [30]. Bcl-2 and MDM2 have been studied in relation to cell survival and cell-cycle regulation [31]. A study of ameloblastoma and ameloblastic carcinoma demonstrated higher MCM2 and MCM3 labeling indices than Ki-67 but found no significant association between marker expression and clinical variables, including recurrence [30]. More recently, a study of unicystic ameloblastoma reported greater Bcl-2 and MDM2 expression and higher Ki-67 indices in intraluminal and/or mural variants and recurrent cases than in purely luminal lesions [31]. These findings illustrate the biological information provided by immunohistochemistry while showing why expression differences require separate evaluation as predictors of clinical outcomes. The present study contributes a PRAME expression profile together with formal assessment of interobserver agreement. It does not establish superior performance over other candidate markers, and the predominance of high extent scores leaves its ability to distinguish clinically different tumor subsets uncertain. Studies evaluating PRAME alongside proliferation and survival markers in the same cases, with standardized scoring and adequate longitudinal follow-up, are needed to determine whether it adds information beyond established clinicopathological factors.

The high frequency of 3+ extent scores should not be interpreted as diagnostic specificity. Diffuse nuclear PRAME staining is useful in selected melanocytic diagnostic settings [13], but a systematic survey of more than 5800 tumors demonstrated expression across diverse epithelial and nonepithelial neoplasms [14]. No non-neoplastic odontogenic epithelium was available in the examined material, and no comparative odontogenic tissue group was included. Melanoma served as a technical positive control rather than a biological comparator for odontogenic tissues. The present findings therefore establish the frequency of PRAME immunoreactivity in the sampled ameloblastomas but cannot determine whether expression is enriched relative to normal odontogenic tissues or reflects an odontogenic differentiation pattern. Comparative assessment of normal odontogenic epithelium, odontogenic cysts, other benign odontogenic tumors, and ameloblastic carcinoma is needed to evaluate potential diagnostic specificity.

Reproducibility is a principal strength of the present analysis. High interobserver agreement for extent and intensity supports consistent visual assessment within the sampled TMA tissue under the study conditions. Staining intensity was uniform within the assessed samples, which may have facilitated visual grading. Nevertheless, the two discordant intensity ratings indicate that semiquantitative grading remained observer-dependent. Interobserver variability is a recognized challenge in semiquantitative histopathologic assessment, particularly when heterogeneous tumor regions are evaluated, further emphasizing the importance of standardized scoring protocols. The importance of tissue sampling is also illustrated by a study of tumor-infiltrating lymphocyte assessment in triple-negative breast carcinoma, in which interobserver concordance differed between tissue microarrays and whole sections [32]. The present agreement estimates apply to the sampled TMA tissue and do not establish uniform staining throughout each tumor or reproducibility across laboratories. The predominance of negative and highest-category extent scores also limits conclusions about agreement within the intermediate categories. Comparison with whole-tumor sections and independent assessment of a broader range of staining patterns would help establish the generalizability of the scoring framework. Digital image analysis may provide an adjunct to, rather than a replacement for, expert assessment.

PRAME positivity was more frequent in women than in men in this cohort, but the association should be interpreted cautiously. The unadjusted odds ratio was 12.0, with a wide 95% confidence interval of 1.27–113.74, reflecting substantial uncertainty associated with the small PRAME-negative subgroup. Although the unadjusted two-sided Fisher exact *p* value was 0.029, the association did not meet the 0.05 threshold after Holm adjustment across the eight clinicopathological comparisons (adjusted *p* = 0.230). These findings do not establish a biologically defined sex-dependent expression pattern and require independent replication. Large ameloblastoma series generally show little or no clinically meaningful sex predilection [16,17], and no established sex-specific mechanism for PRAME expression in ameloblastoma is known.

All 11 patients with documented recurrence had PRAME-positive tumors; however, this observation requires interpretation in the context of treatment imbalance and sparse subgroup counts. Ten recurrences occurred among 24 conservatively treated patients, compared with one among 13 radically treated patients. Five of the seven PRAME-negative patients underwent radical surgery, leaving only two conservatively treated PRAME-negative patients for comparison. The absence of recurrence in these small negative subgroups therefore does not establish a low underlying recurrence risk, as illustrated by the wide confidence intervals in Table 4. The binary PRAME-recurrence comparison did not meet the conventional significance threshold before or after multiplicity adjustment. An exploratory Firth penalized logistic regression analysis provided a finite estimate of the PRAME-recurrence association after adjustment for surgical approach, but the confidence interval was extremely wide and included the null value. Thus, the adjusted analysis remained inconclusive. Adjustment for surgical approach alone does not account for differences in tumor extent, margin status, or duration of observation. However, the PRAME-negative group did not have a shorter follow-up than the PRAME-positive group (median, 42 vs. 39 months), reducing the likelihood that the absence of documented recurrences in PRAME-negative tumors was simply attributable to a shorter observation period. The PRAME-assessed tissue was not decalcified, including tissue obtained from radical resections, so decalcification of the stained material was not a source of processing differences between the surgical groups. These results neither establish an independent association between PRAME expression and recurrence nor exclude a potentially relevant association requiring evaluation in a larger, appropriately characterized cohort.

A time-to-event design would be preferable to a binary recurrence endpoint because ameloblastoma may recur many years after treatment. The median follow-up of 42 months is informative but does not exclude late recurrence in all patients. Future studies should capture dates of treatment, recurrence, and censoring; estimate recurrence-free survival; and use penalized or exact regression if sparse data persist. Stratification by conventional versus unicystic tumor and by conservative versus radical treatment will be essential.

The PRAME findings should also be placed alongside the established MAPK literature. Activating FGFR2-RAS-BRAF alterations are central to ameloblastoma biology [9,33], but no consistent association between BRAF V600E mutation and recurrence has been demonstrated [10]. Mutation-specific VE1 immunohistochemistry can be useful, although discrepancies with molecular testing have been reported in odontogenic tumors [34,35]. BRAF and MEK inhibitors have produced substantial responses in selected BRAF V600E-mutant ameloblastomas [11,36,37,38,39]; by contrast, PRAME-directed therapy remains investigational [15]. Strong immunohistochemical expression alone is insufficient to establish therapeutic eligibility, which would also depend on molecular expression, antigen processing and presentation, HLA type, immune trafficking, and safety.

This study has several strengths: it evaluates a rare tumor in a clinically annotated cohort, uses independent pathology assessment, distinguishes extent from intensity, quantifies interobserver agreement, and uses exact tests for sparse categorical data. Its main limitations are the retrospective single-cohort design, small sample size, only seven PRAME-negative tumors, imbalanced histologic subgroups, heterogeneous follow-up, absence of major radiologic and margin variables, the limited precision and restricted covariate adjustment of the exploratory regression analysis, and the absence of a time-to-event analysis. Assessment was limited to the selected TMA tissue, and regional variation outside the sampled areas was not evaluated. PRAME was assessed exclusively by immunohistochemistry, without independent confirmation using RNA-based or alternative protein assays, functional experiments, or an odontogenic comparator series.

Multicenter validation should use harmonized pathology review, prespecified scoring thresholds, primary and recurrent specimens, paired samples where available, appropriate odontogenic controls, and molecular confirmation of PRAME. Clinical analyses should focus on recurrence-free survival and adjust for surgery, margins, site, size, histologic type, and follow-up. Integration with BRAF status, proliferation markers, matrix-remodeling pathways, RANKL/OPG, and immune-microenvironment measures may determine whether PRAME adds information beyond established clinicopathologic factors. At present, PRAME is best regarded as a common and reproducibly detectable research biomarker in ameloblastoma, not as a basis for surgical extent, surveillance intensity, or systemic therapy.

## 4. Materials and Methods

### 4.1. Study Design and Case Selection

This retrospective observational study included 37 patients with a histologically confirmed diagnosis of ameloblastoma identified from the archives of Clinic for Maxillofacial Surgery, School of Dental Medicine, University of Belgrade over a 15-year period (2008–2023). Only primary ameloblastomas newly treated during the study period were included; patients presenting with recurrence following treatment before the study period were not included. PRAME immunohistochemistry was performed exclusively on specimens from the initial primary tumor, not on recurrent tumor tissue. Documented recurrence was defined as tumor recurrence occurring during follow-up after surgical treatment of the corresponding primary tumor. Cases were retrieved through a search of clinic database with final inclusion criteria defined as all patients having undergone surgical treatment of histopathologically confirmed ameloblastoma in Laboratory Department of Pathology, School of Dental Medicine, University of Belgrade and exclusion criteria as incomplete documentation such as unavailable duration of follow-up, unknown presence of recurrence or undocumented surgical approach. Archived clinical, operative, follow-up, and histopathologic data were reviewed. Variables for the PRAME-focused analysis were age at diagnosis, sex, maxillary or mandibular site, side, mandibular subsite, histologic type and dominant subtype, surgical approach, reconstruction, recurrence, repeat recurrence, metastasis, and duration of follow-up.

### 4.2. Histopathologic Review

Available hematoxylin-eosin-stained slides and corresponding formalin-fixed, paraffin-embedded tissue blocks were reviewed, to confirm the diagnosis and classify tumors according to the 2022 World Health Organization criteria. Tumors were categorized as conventional or unicystic, and conventional tumors were assigned a dominant histological pattern. No mixed histological patterns were identified. Representative viable tumor areas were selected on hematoxylin-eosin review for tissue microarray (TMA) construction, and sections from the resulting TMA block were used for immunohistochemical analysis.

For radical resection specimens, soft-tissue tumor fragments were separated during routine tissue processing and retained without decalcification for subsequent immunohistochemical analysis. Decalcified tissue was not used for TMA construction or PRAME assessment. No non-neoplastic odontogenic epithelium was available for comparative evaluation in the examined material.

### 4.3. PRAME Immunohistochemistry and Scoring

PRAME immunohistochemistry was performed on TMA sections. A rabbit monoclonal anti-PRAME antibody (ab219650; Abcam, Cambridge, UK) was applied at a dilution of 1:500 using a BOND RX (Leica Biosystems, Nußloch, Germany) instrument and the BOND Polymer Refine Detection system (DS9800; Leica Biosystems, Newcastle, UK). Antigen retrieval was performed using Tris–EDTA buffer at pH 9.0. Sections were incubated with the primary antibody for 30 min at room temperature and counterstained with hematoxylin. Melanoma tissue served as the positive control. For the negative reagent control, the primary antibody was replaced with phosphate-buffered saline.

Two pathologists evaluated the stained sections independently, blinded to clinical information and outcomes, and without access to each other’s scores. For each case, assessment included all available tumor tissue represented in the TMA section rather than selected microscopic fields. Staining extent was recorded as the percentage of tumor cells showing nuclear PRAME immunoreactivity relative to the total number of tumor cells in the assessed tissue. Extent was categorized as follows: score 0, <25%; score 1+, 25% to <50%; score 2+, 50% to <75%; and score 3+, ≥75%. For exploratory clinicopathological comparisons, an extent score above 0, corresponding to nuclear labeling in at least 25% of tumor cells, was classified as PRAME-positive. This operational definition was used for the study’s exploratory comparisons and does not represent a validated diagnostic or prognostic threshold.

Staining intensity was evaluated separately as absent, weak, moderate, or strong. Staining intensity was assessed semiquantitatively by visual evaluation, and no objectively calibrated intensity thresholds were applied. Intensity was uniform within the assessed TMA tumor samples, and no heterogeneous intrasectional intensity patterns requiring selection of a predominant or maximum intensity were encountered.

### 4.4. Interobserver Reproducibility

Interobserver agreement was evaluated separately for PRAME staining extent and intensity using the original paired ratings from the two pathologists. Exact agreement was expressed as the number and percentage of identically classified cases. Unweighted Cohen’s κ and corresponding 95% confidence intervals were calculated for both assessments using asymptotic standard errors; confidence limits extending beyond the allowable range were bounded at −1 and 1 for presentation. Linear- and quadratic-weighted κ statistics were additionally calculated for staining intensity, with categories ordered as absent, weak, moderate, and strong. The anonymized paired observations are provided in Supplementary Table S1.

### 4.5. Statistical Analysis

Categorical variables are presented as counts and percentages. Continuous variables are summarized as mean and standard deviation or median and range, as appropriate. The proportion of tumors classified as PRAME-positive is accompanied by an exact binomial 95% confidence interval. Exploratory 2 × 2 comparisons used two-sided Fisher exact tests because of sparse cell counts; age was compared using an independent-samples t test. The association between sex and PRAME positivity was additionally summarized using an unadjusted odds ratio with a 95% Wald confidence interval calculated on the log-odds-ratio scale.

As a post hoc sensitivity analysis, Holm adjustment was applied to the eight PRAME-clinicopathological comparisons presented in Table 3: sex, age, primary site, histological type, mandibular subsite, side, recurrence, and surgical approach. Both unadjusted and adjusted *p* values are reported. This adjustment addresses the specified family of comparisons in the present manuscript and does not constitute a global correction across other analyses conducted in the broader source dataset. A threshold of *p* < 0.05 was used, but all associations were interpreted as exploratory and considered alongside subgroup sizes, effect estimates, and their uncertainty.

To describe the distribution of documented recurrence across treatment groups, recurrence counts and proportions were tabulated jointly by PRAME status and surgical approach. Radical procedures with and without primary reconstruction were combined. Exact binomial 95% confidence intervals were calculated for the subgroup recurrence proportions. These proportions describe documented recurrence during the available observation period and are not fixed-time recurrence-risk estimates.

An additional post hoc exploratory analysis used Firth’s penalized-likelihood logistic regression to examine the association between PRAME status and documented recurrence, with adjustment for surgical approach. The model was fitted using the grouped binomial counts presented in Table 4 and included PRAME status (positive versus negative) and surgical approach (radical versus conservative) as the only explanatory variables, without interaction terms or automated variable selection. Firth’s method was used to address separation arising from the absence of documented recurrences in the PRAME-negative group. Adjusted odds ratios are reported with 95% profile penalized-likelihood confidence intervals and unadjusted penalized likelihood-ratio *p* values. This analysis evaluated binary documented recurrence during the available observation period, rather than time to recurrence, and was interpreted as a sensitivity analysis rather than prognostic validation.

**Figure 6:**
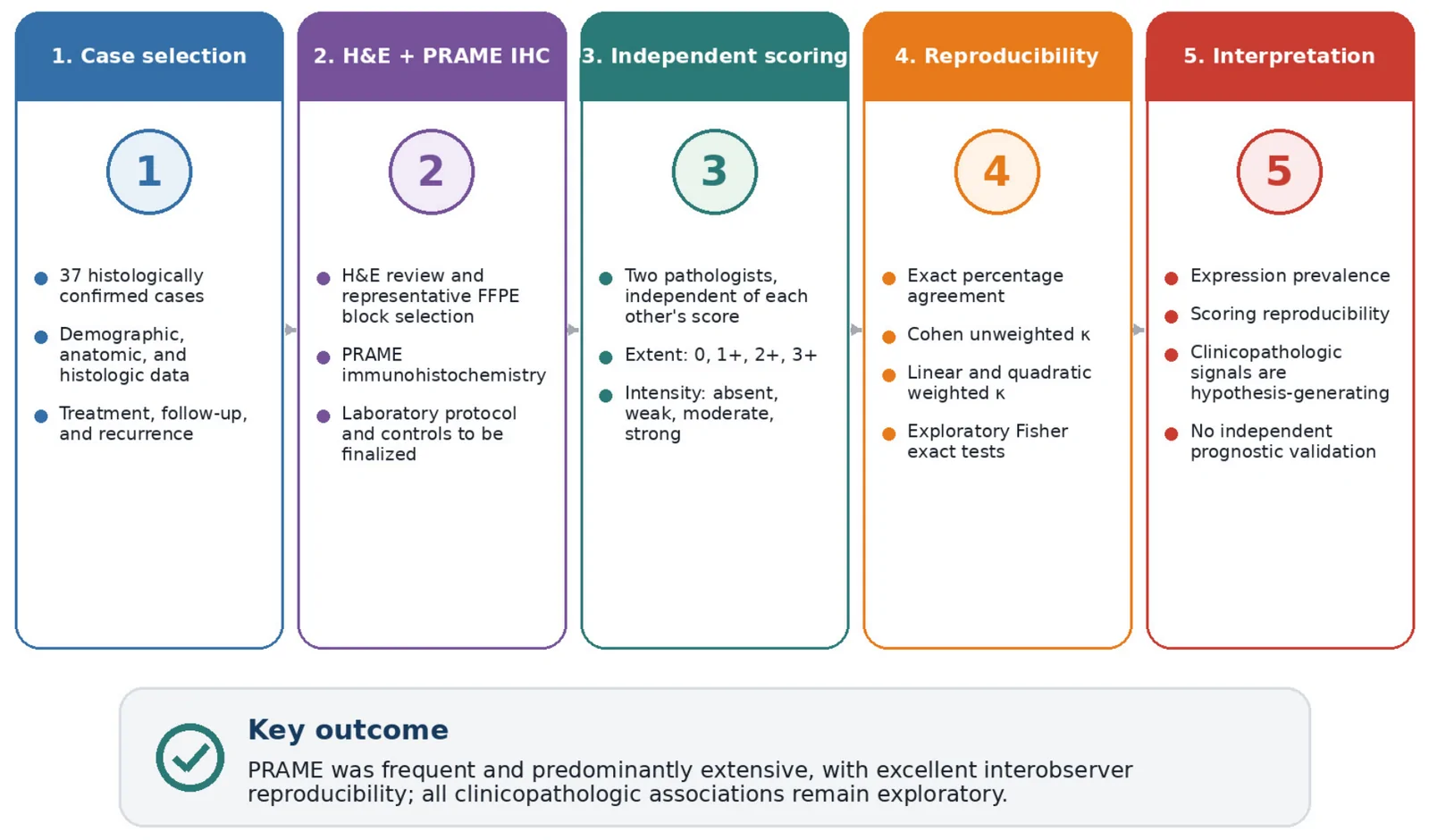
Study design and analytical workflow. Thirty-seven histologically confirmed ameloblastomas underwent clinicopathologic annotation, independent PRAME assessment by two pathologists, reproducibility analysis, and exploratory association testing.

Original analyses were performed using IBM SPSS Statistics version 29.0 (IBM Corp., Armonk, NY, USA). Additional statistical verification and revision analyses were performed using Python version 3.13.5, SciPy version 1.17.0, and statsmodels version 0.14.6. Firth regression and profile penalized-likelihood confidence intervals were implemented in Python using SciPy optimization routines. The overall study design and analytical workflow are summarized in Figure 6.

## 5. Conclusions

This study documents frequent PRAME expression in ameloblastoma and high agreement between two pathologists under the assay conditions evaluated. Its principal contribution is a dataset characterizing PRAME expression and scoring reproducibility, providing a foundation for further investigation of PRAME in odontogenic tumors. The exploratory clinicopathologic findings do not establish diagnostic specificity, independent recurrence prediction, or a role in therapeutic selection. Further evaluation should include independent multicenter cohorts, appropriate odontogenic controls, and recurrence analyses that account for surgical management, tumor characteristics, and duration of follow-up. Such studies are needed to determine whether reproducible PRAME expression translates into clinically useful information.

## Figures and Tables

**Table 1:** Clinicopathologic characteristics of the 37-patient cohort.

Characteristic	Category	Value
Sex	Women	21 (56.8%)
	Men	16 (43.2%)
Age, years	Mean ± SD	41.5 ± 18.9
Primary site	Mandible	33 (89.2%)
	Maxilla	4 (10.8%)
Mandibular subsite	Posterior	27/33 (81.8%)
	Anterior	6/33 (18.2%)
Side	Left	21 (56.8%)
	Right	16 (43.2%)
Histologic type	Conventional	34 (91.9%)
	Unicystic	3 (8.1%)
Conventional subtype	Follicular	31/34 (91.2%)
	Plexiform	3/34 (8.8%)
Surgical approach	Conservative	24 (64.9%)
	Radical	13 (35.1%)
Reconstruction	Among radical procedures	6/13 (46.1%)
Recurrence	Yes	11 (29.7%)
	No	26 (70.3%)
Repeat recurrence	Among recurrent tumors	1/11 (9.1%)
Metastasis	Yes	1 (2.7%)
Follow-up, months	Median (range)	42 (2–120)

Values are *n* (%) unless otherwise specified. Percentages for mandibular subsite, conventional subtype, reconstruction, and repeat recurrence use the corresponding subgroup denominator.

**Table 2:** PRAME expression and interobserver reproducibility.

Assessment	Category or Statistic	Pathologist 1	Pathologist 2
Extent score	0	7 (18.9%)	7 (18.9%)
	1+	1 (2.7%)	0 (0.0%)
	2+	0 (0.0%)	1 (2.7%)
	3+	29 (78.4%)	29 (78.4%)
Intensity	Absent	7 (18.9%)	7 (18.9%)
	Weak	1 (2.7%)	0 (0.0%)
	Moderate	8 (21.6%)	7 (18.9%)
	Strong	21 (56.8%)	23 (62.2%)
Extent agreement	Exact agreement	36/37 (97.3%)	Cohen kappa = 0.923
Intensity agreement	Exact agreement	35/37 (94.6%)	Unweighted kappa = 0.905

The 95% CI for the unweighted intensity kappa was 0.778–1.000. Extent scores were concordant in 36 of 37 cases (97.3%), with one 1+ versus 2+ disagreement.

**Table 3:** Clinicopathologic characteristics according to PRAME status.

Characteristic	PRAME-Negative	PRAME-Positive	Unadjusted *p* Value	Holm-Adjusted *p* Value
Patients, n	7	30	-	-
Women	1 (14.3%)	20 (66.7%)	0.029	0.230
Age, years	48.6 ± 16.2	39.8 ± 19.3	0.276	1.000
Primary site: mandible	6 (85.7%)	27 (90.0%)	1.000	1.000
Conventional histological type	7 (100.0%)	27 (90.0%)	1.000	1.000
Posterior mandibular site *	4/6 (66.7%)	23/27 (85.2%)	0.295	1.000
Left side	3 (42.9%)	18 (60.0%)	0.437	1.000
Documented recurrence	0 (0.0%)	11 (36.7%)	0.080	0.506
Radical surgery	5 (71.4%)	8 (26.7%)	0.072	0.506

Values are n (%) unless otherwise specified; age is presented as mean ± standard deviation. Percentages are calculated within each PRAME group. * Posterior mandibular site is calculated only among patients with mandibular tumors, using the subgroup denominators shown. Categorical *p* values are from two-sided Fisher exact tests; the age *p* value is from an independent-samples *t* test. Holm adjustment was applied as a post hoc sensitivity analysis across the eight comparisons in this table. All association analyses were exploratory.

**Table 4:** Documented recurrence according to PRAME status and surgical approach.

Surgical Approach	PRAME Status	Patients, n	Documented Recurrence, n/N (%)	Exact 95% CI for Recurrence Proportion
Conservative	Positive	22	10/22 (45.5%)	24.4–67.8%
Conservative	Negative	2	0/2 (0.0%)	0.0–84.2%
Radical, including reconstruction	Positive	8	1/8 (12.5%)	0.3–52.7%
Radical, including reconstruction	Negative	5	0/5 (0.0%)	0.0–52.2%

Radical surgery includes procedures with and without primary reconstruction. Percentages use the denominator within each PRAME–treatment subgroup. Confidence intervals are exact binomial intervals. These are observed recurrence proportions during the available observation period, not fixed-time recurrence-risk estimates. The table is descriptive and does not adjust for differences in observation time or other potential confounders.

## Data Availability

The data supporting the findings of this study are available from the corresponding author upon reasonable request. The data are not publicly available due to privacy and ethical restrictions related to patient data.

## Supplementary Materials

The following supporting information can be downloaded at: https://www.mdpi.com/article/10.3390/ijms27219771/s1.
